# Supplementary figures and images for: Curd, seed yield and disease resistance of cauliflower are enhanced by oligosaccharides
Source: PeerJ. 2024 Mar 25;12:e17150. doi: 10.7717/peerj.17150 (PMC10977091; doi:10.7717/peerj.17150)

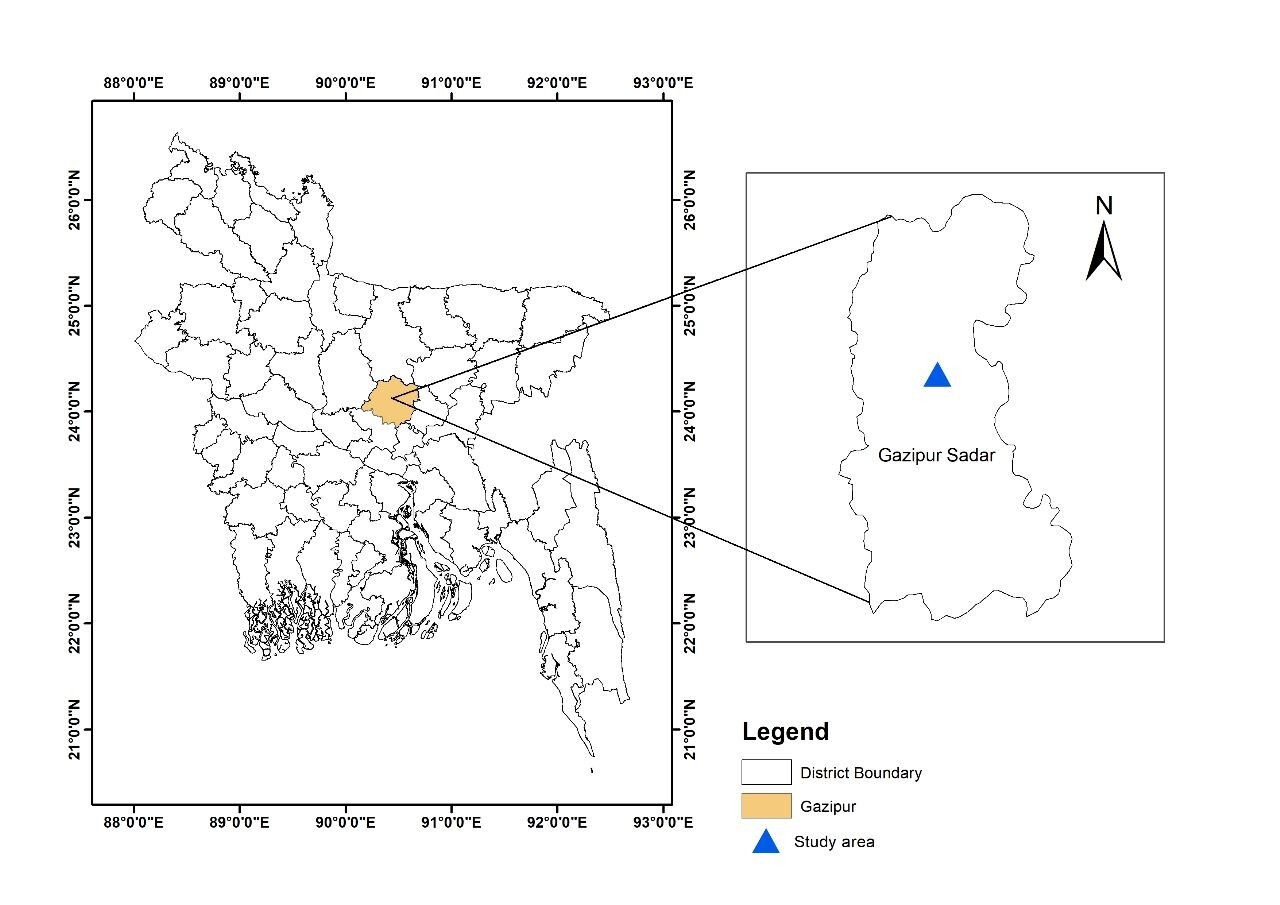

Supplement: Supplemental Information 1 [file peerj-12-17150-s001.jpg]
