# Supplementary material for: Curd, seed yield and disease resistance of cauliflower are enhanced by oligosaccharides
Source: PeerJ. 2024 Mar 25;12:e17150. doi: 10.7717/peerj.17150 (PMC10977091; doi:10.7717/peerj.17150)
Supplement: Supplemental Information 2 [file peerj-12-17150-s002.docx]

**Table S1:**

Physical and chemical properties of initial soil in the experimental field, BSMRAU, Gazipur, Bangladesh.

| Sl. No. | Soil properties | Status | Critical value |
| --- | --- | --- | --- |
| 1 | Soil texture | Clay loam | - |
| 2 | Soil PH | 6.2 | - |
| 3 | Bulk density (g/cc) | 1.4 | - |
| 4 | CEC (meq 100 g^-1^) | 10.5 | - |
| 5 | Organic carbon (%) | 0.86 | - |
| 6 | Total N (%) | 0.09 | 0.12 |
| 7 | Available P (μg/g) | 10.22 | 7.00 |
| 8 | Exchangeable K (meq 100 g^-1^) | 0.07 | 0.12 |
| 9 | Available S (μg/g) | 13.5 | 10.00 |
| 10 | Available Zn (μg/g) | 0.9 | 0.60 |
| 11 | Available B (μg/g) | 0.28 | 0.20 |
