# Supplementary material for: Curd, seed yield and disease resistance of cauliflower are enhanced by oligosaccharides
Source: PeerJ. 2024 Mar 25;12:e17150. doi: 10.7717/peerj.17150 (PMC10977091; doi:10.7717/peerj.17150)
Supplement: Supplemental Information 3 — *Monthly total, **Monthly Average, a provided by: Department of Agricultural Engineering, BSMRAU. ahttp://bsmrau.edu.bd/age/weather-data/ [file peerj-12-17150-s003.docx]

**Table S2:**

Climatic condition of Bangabandhu Sheikh Mujibur Rahman Agricultural University (BSMRAU), Gazipur, Bangladesh during the experiments^a^

| Month/Year | ** Air Temperature (^o^C) | | | ** Soil Temperature (^o^C) at 30 cm Depth | **Humidity  (%) | *Rainfall  (mm) | *Evaporation (mm) |
| --- | --- | --- | --- | --- | --- | --- | --- |
|  | Maximum Average | Minimum Average | Average |  |  |  |  |
| September21 | 34.35 | 27.18 | 30.77 | 27.43 | 83.87 | 85.71 | 113.96 |
| October21 | 33.63 | 25.48 | 29.56 | 27.10 | 84.58 | 100.65 | 107.12 |
| November21 | 30.47 | 18.32 | 24.39 | 23.77 | 91.03 | 11.36 | 73.55 |
| December21 | 26.55 | 14.81 | 20.68 | 18.58 | 88.32 | 69.97 | 54.27 |
| January22 | 24.87 | 13.45 | 19.16 | 16.00 | 87.58 | 8.77 | 45.39 |
| February22 | 26.11 | 12.84 | 19.47 | 17.71 | 87.36 | 30.68 | 57.75 |
| March22 | 33.95 | 19.73 | 26.84 | 24.45 | 84.19 | 12.99 | 106.01 |
| April22 | 34.67 | 26.25 | 30.46 | 27.10 | 84.07 | 55.52 | 138.23 |

| *Monthly total, **Monthly Average, ^a^ provided by: Department of Agricultural Engineering, BSMRAU. ^a^ http://bsmrau.edu.bd/age/weather-data/ |
| --- |
