# Supplementary material for: Curd, seed yield and disease resistance of cauliflower are enhanced by oligosaccharides
Source: PeerJ. 2024 Mar 25;12:e17150. doi: 10.7717/peerj.17150 (PMC10977091; doi:10.7717/peerj.17150)
Supplement: Supplemental Information 4 — XAll treatments were applied at 15, 30, 45 and 60 days after transplanting @ 50 mg.L-1 except control (sterile water), COS = chitosan oligosaccharide, OGA= oligo galacturonic acid, AOS = alginate oligo saccharide, values are as the means ± SD in two independent measurements. LSD = least significance difference value at 0.05%, NS = not significant, asterisks indicate significant differences (*P < 0.05, **P < 0.01). Yng = nanogram, g-1 = per gram, FW = fresh weight basis, SPAD = Soil Plant Development Analysis. [file peerj-12-17150-s004.docx]

**Table S3.**

SPAD value, chlorophyll content and disease index in leaves of BU cauliflower 1under different oligosaccharides treatments.

| Treatment^X^ | Early Season (15 September 2021)^Y^ | | | Mid-Season (15 October 2021) | | |
| --- | --- | --- | --- | --- | --- | --- |
|  | SPAD value (%) | Chlorophyll (ng.g^-1^ FW) | Disease index (%) | SPAD | Chlorophyll (ng.g^-1^ FW) | Disease index (%) |
| Control | 0.65±0.1 | 1.70±0.1 | 75.18±1.3 | 0.63±0.1 | 1.60±0.1 | 75.66±0.3 |
| COS 50 | 3.29±0.5 | 2.50±0.5 | 62.78±5.3 | 3.27±0.5 | 2.45±0.5 | 61.71±4.9 |
| OGA 50 | 2.56±0.6 | 2.10±0.3 | 67.09±8.1 | 2.52±0.6 | 2.03±0.3 | 67.75±8.0 |
| AOS 50 | 2.74±1.0 | 2.46±0.3 | 60.41±3.0 | 2.76±1.0 | 2.54±0.2 | 59.18±2.3 |
| Level of significant | ** | NS | * | ** | * | ** |
| LSD (0.05%) | 1.50 | - | 11.10 | 1.07 | 1.77 | 7.62 |
| ^X^All treatments were applied at 15, 30, 45 and 60 days after transplanting @ 50 mg.L^-1^ except control (sterile water), COS = chitosan oligosaccharide, OGA= oligo galacturonic acid, AOS = alginate oligo saccharide, values are as the means ± SD in two independent measurements. LSD = least significance difference value at 0.05%, NS = not significant, asterisks indicate significant differences (*P < 0.05, **P<0.01). ^Y^ng = nanogram, g^-1^ = per gram, FW = fresh weight basis, SPAD = Soil Plant Development Analysis. | | | | | | |
